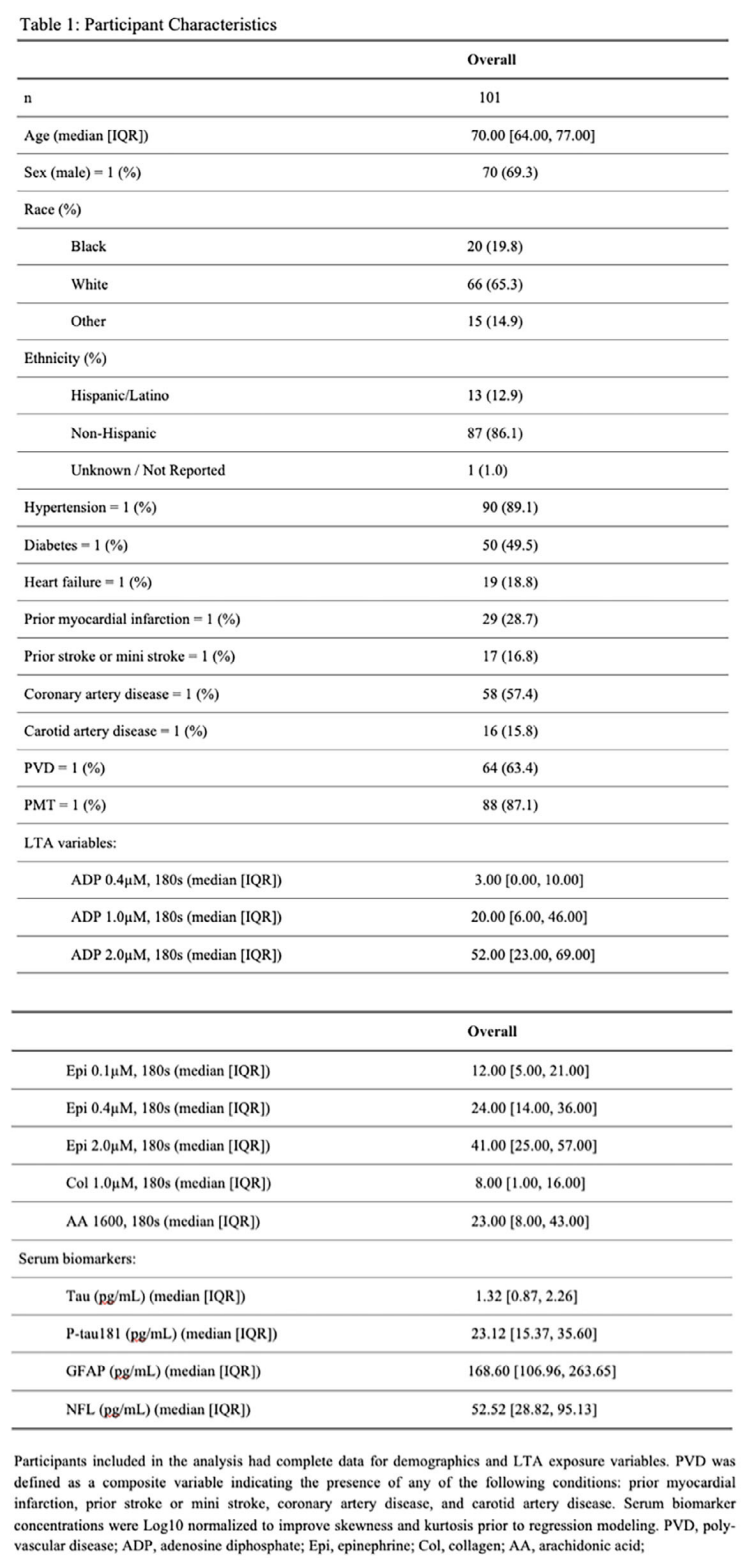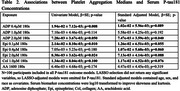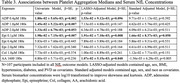# Elevated Platelet Aggregation Associates with Increased Alzheimer's Disease Blood Biomarker Concentrations in Patients with Peripheral Artery Disease

**DOI:** 10.1002/alz70855_107122

**Published:** 2025-12-25

**Authors:** Tovia Jacobs, Jaime Ramos Cejudo, Mark He, Matthew Muller, Luisa F Figueredo, Jeffrey S Berger, Ricardo S. Osorio

**Affiliations:** ^1^ NYU Grossman School of Medicine, New York, NY, USA; ^2^ New York University (NYU) Grossman School of Medicine, New York City, NY, USA; ^3^ New York University School of Medicine, New York, NY, USA

## Abstract

**Background:**

Cardiovascular risk factors are among the most significant contributors to dementia, yet the biological mediators involved in this relationship need to be clarified. Prior research shows that elevated platelet aggregation is associated with an increased risk of dementia. However, comorbidities that influence platelet activity and serve as risk factors for Alzheimer's disease (AD) and related dementias (AD/ADRD) complicate this association, particularly in individuals with high vascular burden who do not have dementia. To address this, we leveraged the NIH‐funded Platelet Activity and Cardiovascular Events (PACE) in patients with peripheral artery disease (PAD) and examined the relationship between platelet aggregation and AD biomarkers.

**Method:**

We assessed associations between platelet aggregation measured by light transmission aggregometry (LTA) and concentrations of phosphorylated tau (*p*‐tau181), total tau, neurofilament light (NfL), and glial fibrillary acidic protein (GFAP) in serum samples using SIMOA (Quanterix). Quantile regression models, using median cutoffs (τ=0.5), were employed to evaluate the relationships across three levels of adjustment: (1) univariate, (2) LASSO‐selected covariates, and (3) adjusted by age, sex, race, and the use of platelet modifying treatments (PMT), such as aspirin or clopidogrel.

**Result:**

Of the 125 participants included in the analysis, 101 had complete measures of LTA exposures and all AD sera biomarkers. The median age of participants was 70 [Q1=64, Q3=77], 69.3% were male, 63.4% had poly‐vascular disease (PVD), and 87.1% were on PMT (Table 1). Elevated platelet aggregation in response to submaximal doses of ADP and Epinephrine (Epi) associated with increased *p*‐tau181 (Table 2) and NfL levels (Table 3). Associations between platelet aggregation and tau and GFAP were not observed.

**Conclusion:**

Although limited in sample size, our study identified associations of platelet aggregation with markers of AD pathology (*p*‐tau181) and neurodegeneration (NfL) in PAD patients without dementia. Future studies in a larger cohort are needed to further investigate this relationship and the potential role of platelet aggregation as a mediator of AD pathology and neuroinflammatio